# *Faecalibacterium prausnitzii* in Differentiated Thyroid Cancer Patients Treated with Radioiodine

**DOI:** 10.3390/nu15122680

**Published:** 2023-06-08

**Authors:** Ana Fernandes, Ana Oliveira, Ana Luísa Carvalho, Raquel Soares, Pedro Barata

**Affiliations:** 1 Department of Nuclear Medicine, Centro Hospitalar Universitário de São João, E.P.E., 4200-319 Porto, Portugal; ana.ccoliveira@chsj.min-saude.pt (A.O.); a.carvalho@chsj.min-saude.pt (A.L.C.); 2 Department of Biomedicine, Faculdade de Medicina da Universidade do Porto, 4200-319 Porto, Portugal; raqsoa@med.up.pt; 3 i3S—Instituto de Investigação e Inovação em Saúde, Universidade do Porto, 4200-135 Porto, Portugal; pbarata@ufp.edu.pt; 4 Department of Pharmaceutical Science, Faculdade de Ciências da Saúde da Universidade Fernando Pessoa, 4249-004 Porto, Portugal; 5 Department of Pathology, Centro Hospitalar Universitário do Porto, 4099-001 Porto, Portugal

**Keywords:** *Faecalibacterium prausnitzii*, thyroid cancer, radioiodine therapy, gut microbiota, metagenomic sequencing

## Abstract

Background: *Faecalibacterium prausnitzii*, one of the most important bacteria of the human gut microbiota, produces butyrate (a short-chain fatty acid). Short-chain fatty acids are known to influence thyroid physiology and thyroid cancer’s response to treatment. We aimed to analyze the relative abundance of *Faecalibacterium prausnitzii* on the gut microbiota of differentiated thyroid cancer patients compared to controls and its variation after radioiodine therapy (RAIT). Methods: Fecal samples were collected from 37 patients diagnosed with differentiated thyroid cancer before and after radioiodine therapy and from 10 volunteers. The abundance of *F. prausnitzii* was determined using shotgun metagenomics. Results: Our study found that the relative abundance of *F. prausnitzii* is significantly reduced in thyroid cancer patients compared to volunteers. We also found that there was a mixed response to RAIT, with an increase in the relative and absolute abundances of this bacterium in most patients. Conclusions: Our study confirms that thyroid cancer patients present a dysbiotic gut microbiota, with a reduction in *F. prausnitzii’s* relative abundance. In our study, radioiodine did not negatively affect *F. prausnitzii*, quite the opposite, suggesting that this bacterium might play a role in resolving radiation aggression issues.

## 1. Introduction

*Faecalibacterium prausnitzii* (*F. prausnitzii; NCBI:txid853*) is a butyrate-producing bacterium and one of the most important species in the human gut microbiota [1,2].

Previously known as *Fusobacterium prausnitzii* [3,4], *F. prausnitzii* belongs to the Clostridia cluster IV [5,6,7] and to the *Faecalibacterium* genus. It is an anaerobic, non-spore-forming, and non-motile Gram-positive bacterium. *F. prausnitzii* is one of the most common species of the healthy gut microbiota, representing over 5% (5–15%) of the total fecal microbiota in adults [1,3,4,8,9]. Its most important functions include the synthesis of butyrate (an essential short-chain fatty acid (SCFA)), the production of energy for the colonocytes, and the production of anti-inflammatory metabolites such as salicylic acid. *F. prausnitzii* may prevent inflammation by inducing the production of IL-10 and inhibiting the synthesis of pro-inflammatory cytokines (IL-6 and IL-12) [10,11]. It can also produce salicylic acid [12] and restore serotonin [13] to normal levels [11]. A reduction in *F. prausnitzii* has been reported in several diseases characterized by gut dysbiosis, including Crohn’s disease [14], ulcerative colitis [15], celiac disease [16], and obesity [17]. Importantly, *F. prausnitzii* has been suggested to serve as a biomarker for monitoring thyroid health [18].

Butyrate is known to suppress cancer cells’ activity and life cycle [19], supports a healthy intestinal environment, and is the preferred energy source for normal colonocytes [20]. It also presents anti-inflammatory properties [21,22,23,24] and pleiotropic effects in the intestinal cell life cycle, among other beneficial health effects [3]. As with other dietary antioxidants that promote the proliferation of beneficial gut microbiota, butyrate has been used as a dietary supplement.

Recently, finding butyrate-producing bacteria depletion in multiple diseases (mainly colorectal cancer (CRC) and inflammatory bowel diseases) [25] has prompted the hypothesis of considering them as new-generation probiotics [26].

Thyroid cancer is the most common head and neck malignancy [27]. According to the International Agency for Research on Cancer, it is the fifth most common cancer worldwide [28]. Differentiated thyroid cancers of follicular cell origin (DTC) are the most frequent thyroid cancers, representing more than 90%. Patients with DTC are frequently treated with radioiodine (I-131) therapy (RAIT), consisting of the systemic administration of high doses of I-131 for the ablation of thyroid tissue remnant after surgery for the treatment of recurrence or metastatic disease [29]. Changes in the gut microbiota composition have been associated with thyroid cancer [30,31,32,33]. Short-chain fatty acids, especially butyrate, can inhibit histone deacetylase and activate sodium/iodide symporter (NIS) expression in thyroid cancer cells, thereby enhancing iodine uptake [34]. Additionally, previous research has revealed that SCFAs may act as epigenetic modifiers interfering with NIS expression and iodine uptake [35] and, therefore, affecting the success of therapy.

Ionizing radiation (IR) is a type of energy capable of removing electrons from atoms and molecules due to its energy and penetrating capacity. DNA is considered the critical target for radiation damage; however, proteins, lipids, and metabolites may also have their structure altered, affecting their function or even causing their destruction [36,37].

Ionizing radiation is known to damage the intestinal epithelial barrier and mucus layer, leading to the translocation of bacteria that can trigger an inflammatory response. The resulting dysbiosis can influence both local and systemic immune responses [38]. Both systemic radiation delivery via radiopharmaceuticals (including RAIT) and radiotherapy use ionizing radiation. However, the treatments present major differences concerning dosimetry, dose rate, linear energy transfer, duration of treatment delivery, and target volume, among other parameters. The variety of conditions induces distinct radiation-induced biological responses, thus, making it almost impossible to extrapolate from radiotherapy to nuclear medicine [36,39,40]. Moreover, although there are studies on the changes in the abundance of gut microbiota bacteria, including butyrate-producing bacteria, after abdominal and pelvic radiotherapy [41,42,43,44,45], we could not retrieve any data regarding the effect of RAIT. 

I-131 has been used for both the diagnosis and treatment of benign and malign thyroid diseases since 1941. It consists of the systemic administration of sodium or potassium iodide for selective irradiation of thyroid tissue. After oral administration, I-131 is absorbed rapidly in the upper gastrointestinal tract and is distributed after entering the bloodstream [29]. I-131 is excreted mainly through the urinary system, but a small quantity is secreted from the blood into the colon. Some early effects, mostly transient, may include gastrointestinal symptoms, namely diarrhea, indicating that I-131 might alter the gut microbiota homeostasis [29].

Due to its recognized importance and relation to thyroid cancer, in this prospective study, we specifically evaluated the changes in the abundance of the most important butyrate-producing bacterium, *Faecalibacterium prausnitzii*, after I-131 treatment in 37 differentiated thyroid cancer patients. Additionally, we compared the gut microbiota composition of the thyroid cancer patients with ten volunteers. To do this, we compared fecal samples collected from patients two to three days before treatment and eight to ten days after treatment and compared the samples from the thyroid cancer patients with the samples from volunteers. Understanding the impact of radioiodine therapy on this main bacterium will allow us to predict and minimize side effects by embracing prophylactic/therapeutic attitudes.

## 2. Materials and Methods

### 2.1. Study Population and Treatment

In this prospective cohort study, stool samples from patients with differentiated thyroid cancer were collected to investigate the relative abundance of *F*. *prausnitzii* before and after radioiodine therapy.

We included consecutive patients with differentiated thyroid cancer treated with RAIT at the Centro Hospitalar e Universitário de São João, E.P.E, between February 2019 and April 2021.

Patients were eligible for enrolment if they met the following inclusion criteria: histologically confirmed differentiated thyroid carcinoma, age 18 years or older, and had undergone thyroidectomy. Patients with previous radioiodine treatment or patients incapable of providing consent were excluded. Patients with recent treatment with antibiotics, steroids, immune suppressants, and pre- or pro-biotics were also excluded.

The patients were asked to provide fecal samples within 1 to 2 days before the RAIT and 8 to 10 days post-treatment. The dose rate of the neck and abdomen from each patient at a distance of one meter and the dose rate from the sample at a distance of one centimeter were measured with a detector (Victoreen 190).

Additionally, ten volunteers provided fecal samples to serve as controls. Exclusion criteria included a history of thyroid pathologies, previous nuclear medicine treatments or radiotherapy, and treatments with antibiotics, steroids, or immunosuppressors six months prior to the study.

This work was approved by the Ethics Committee of the Centro Hospitalar e Universitário de São João, E.P.E. and the Faculdade de Medicina da Universidade do Porto (FMUP) on 27th February 2018 (number: 52/18). Furthermore, all subjects gave written informed consent in accordance with the Declaration of Helsinki.

### 2.2. Sample Collection

Patients collected stool samples at two different time points. The first was 1 to 2 days before the treatment (pre-RAIT samples), and the second was collected 8 to 10 days after the treatment (post-RAIT samples). In addition, patients completed a questionnaire at each time point to establish bowel patterns, concomitant medication, and dietary changes. All samples were collected into a sterile plastic container (VWR Specimen Containers, PP) and stored at the laboratory at −80 °C until further processing.

### 2.3. DNA Extraction and Sequencing

DNA from fecal samples was obtained utilizing the Invitrogen™ PureLink™ Microbiome Kit following the manufacturer’s proposed methodology.

The Illumina Nextera XT DNA Library Preparation Kit (San Diego, CA, USA) was used to prepare DNA Libraries and IDT Unique Dual Indexes, with total DNA input of 1ng. Genomic DNA was fragmented with a proportional amount of fragmentation enzyme. To each sample, unique dual indexes were added, and 12 cycles of PCR were run to construct libraries which were purified using AMpure magnetic beads (Beckman Coulter) and eluted in QIAGEN EB buffer. The DNA libraries were quantified using fluorometry using the Qubit 4 and the Qubit dsDNA HS Assay Kit (Thermo Fisher Scientific, Wilmington, DE, USA). Libraries were then sequenced on the Illumina NovaSeq platform 2 × 150 bp.

### 2.4. Metagenomic Profiling and Statiscal Analysis

Unassembled sequencing reads were directly analyzed using the CosmosID bioinformatics platform (CosmosID Inc., Rockville, MD, USA) for multi-kingdom microbiome analysis to quantify the relative and absolute abundances of the bacteria.

A high-performance data-mining k-mer algorithm was used. This algorithm disambiguates millions of short sequence reads into genomes that engender particular sequences. The pipeline includes two separable comparators: one consists of a pre-computation phase for reference databases, and the other is a per-sample computation.

The inputs to the pre-computation phase are databases of reference genomes that are continuously curated by CosmosID, and the output is a phylogeny tree of microbes combined with sets of biomarkers uniquely associated with distinct branches and leaves of the tree.

The second computational phase consists of searching the millions of short sequence reads, or, alternatively, contigs, from draft de novo assemblies against the biomarkers sets. This allows highly precise and sensitive detection and taxonomic classification of the NGS microbial reads. The obtained statistics are then analyzed to return the fine-grain taxonomic and relative abundance estimates for the microbial NGS datasets. These results are filtered using a threshold based on an internal statistical score, determined by analysis of multiple metagenomes, to exclude false positive identifications. The same approach is applied to enable the sensitive and accurate detection of genetic markers for virulence and resistance to antibiotics.

Assessment of significant differences in the alpha diversity was determined by the Wilcoxon rank-sum test. Alpha diversity was estimated using three indexes: Chao1 for microbial species richness and the Simpson and Shannon indexes for biodiversity and observed species. Additionally, permutational multivariate analysis of variance (PERMANOVA) was used for statistical significance testing of beta dissimilarities between selected groups. Beta diversity was assessed using Bray–Curtis distance-based non-metric multidimensional scaling analysis and the Jaccard dissimilarity index through 999 permutations. Diversity analyses were performed using species taxonomy level. A taxa bar plot was performed to distinguish discrepant abundant species between the different taxa of the groups, from the phylum to species level. To further explore key phylotypes that may contribute to the observed differences in microbial communities between cohorts, linear discriminant analysis (LDA) effect size (LEfSe) was performed to estimate differentially abundant features with biological consistency and statistical significance [46], with an LDA threshold value of >3.0. Statistical significance was considered for *p* < 0.05 values.

Statistical analysis was performed in R v4.1.2. The normality of the relative abundance difference distribution was tested using the Shapiro–Wilk test and by observing the histogram. Differences between relative bacterial abundance pre- and post-RAIT were tested using the Wilcoxon signals rank. Aiming to investigate whether there were variables related to the differences in the relative abundance of each bacterium prior- and post-administration of the radiopharmaceutical, several linear regression models were developed and interpreted.

## 3. Results

### 3.1. Study Population

A total of 37 patients with differentiated thyroid cancer of follicular cell origin met the inclusion criteria, providing 74 samples collected during the study period. In total, 532,242,746 reads with an average of 7,192,470 reads per sample were generated after initial quality filtering (median length: 150 bp).

The median age of the 37 included patients (11 males, 26 females) was 51 ± 17 years (range: 25–81). All patients received RAIT, 7 with 1110 MBq, 22 with 3700 MBq, and 8 with 5550 MBq. One patient had follicular thyroid carcinoma, and the rest had papillary thyroid carcinoma. Basic demographics and clinical characteristics of the study population are presented in Table 1. One patient used thyrotropin alfa, and all others induced hypothyroidism by suspending hormone substitution for four weeks. None had inflammatory intestinal diseases. The volunteers had a mean age of 42 ± 15 years, 4 males and 6 females.

### 3.2. Gut Microbiota Diversity, Richness, and Taxonomic Distribution from Thyroid Cancer Patients before Treatment Compared with Healthy Controls

**Figure 1:**
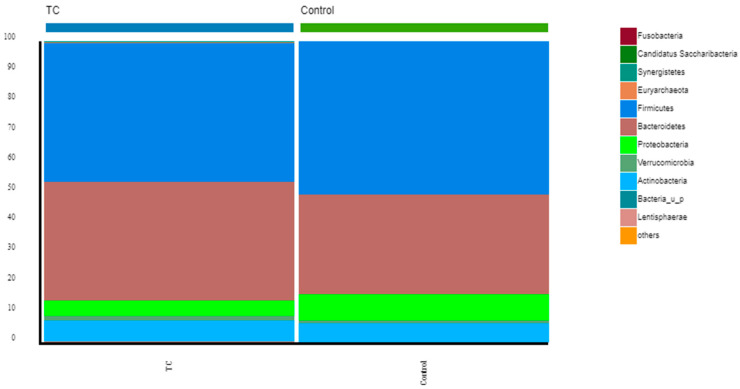
Comparison of bacterial communities between thyroid cancer patients and controls. Stacked bar chart representing the relative abundance of the most frequent phyla from the thyroid cancer patients before RAIT compared with the controls.

At the phylum level, the two prominent phyla were found to be Bacteroidetes (39.26% in thyroid cancer patients; 33.31% in controls) and Firmicutes (46.25% in thyroid cancer; 50.78% in controls). The two represented over 80% of the total phyla (Figure 1 and Table S1 from the Supplementary Material).

**Figure 2:**
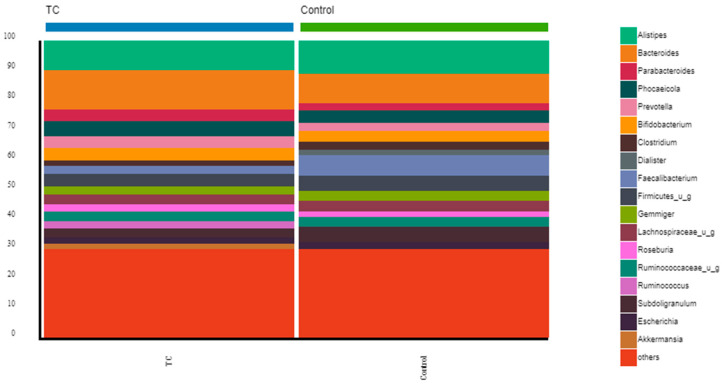
Comparison of bacterial communities between thyroid cancer patients and controls. Stacked bar charts represent the relative abundance of the most frequent genera from the thyroid cancer patients before RAIT and volunteers. Others represent the bottom 30% of the less abundant genera.

At the genus level, the genera that represented more than 5% in thyroid cancer patients were *Bacteroides* (13.25%), *Alistipes* (10.03%), and *Phocaeicola* (5.11%); in the volunteers, they were *Alistipes* (11.04%), *Bacteroides* (10.00%), *Faecalibacterium* (7.06%), and *Subdoligranulum* (5.03%) (Figure 2, and Tables S2 and S3 from the Supplementary Materials).

**Figure 3:**
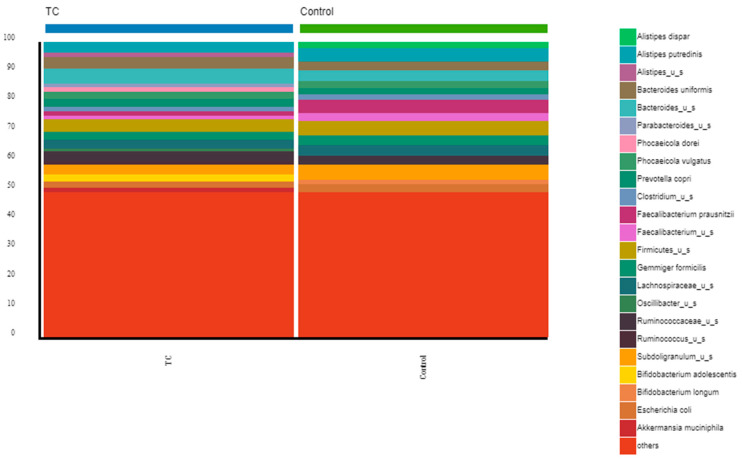
Comparison of bacterial communities between thyroid cancer patients and controls. Stacked bar chart representing the relative abundance at the species level from the thyroid cancer patients before RAIT and controls. Others represent the bottom 50% of the less abundant genera.

Finally, at the species level, the most abundant species (above 4%) in thyroid cancer patients were *Bacteroides_u_s* (5.15%) and *Firmicutes_u_s* (4.23%); in the volunteers, they were *Subdoligranulum_u_s* (5.00%), *Firmicutes_u_s* (4.99%), *Faecalibacterium prausnitzii* (4.50%), and *Alistipes putredinis* (4.39%) (Figure 3, and Tables S4 and S5 from the Supplementary Materials).

In the present study, alpha diversity, evaluated using Chao1 and the Simpson and Shannon indexes, was not significantly different between thyroid cancer patients and control sets of samples (*p* = 0.483, *p* = 0.815, and *p* = 0.406, respectively). Regarding beta diversity, no separating trends between the bacterial communities in pre- and post-treatment groups were observed in terms of Jaccard distance and Bray–Curtis dissimilarity (*p* = 0.322 and *p* = 0.110, respectively).

### 3.3. The Relative Abundance of F. prausnitzii in Differentiated Thyroid Cancer Patients Is Decreased Compared with Controls

**Figure 4:**
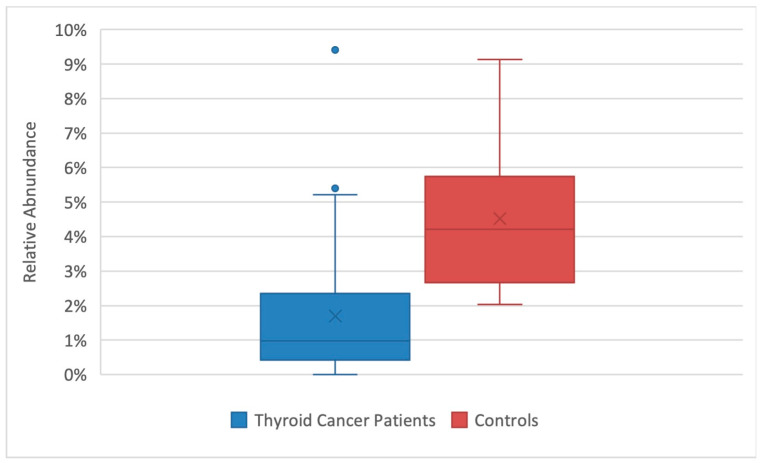
Comparison of *F. prausnitzii’s* relative abundance between thyroid cancer patients pre-RAIT samples and controls. Box-plot representing the relative abundance of *F. prausnitzii* in thyroid cancer patients (blue) and controls (red). Significant differences were found between the relative abundances, *p* = 0.02.

**Figure 5:**
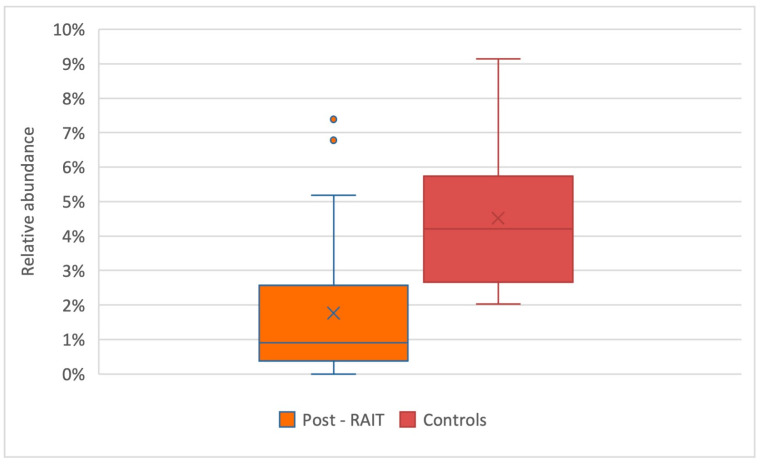
Comparison of *F. prausnitzii’s* relative abundance between thyroid cancer patients post-RAIT samples and controls. Box-plot representing the relative abundance of *F. prausnitzii* in post-RAIT samples from thyroid cancer patients (orange) and controls (red). Significant differences were found between the relative abundances, *p* = 0.002.

In our population, we observed significant differences between the mean relative abundance of *F. prausnitzii* from control compared to pre-RAIT samples (*p* = 0.02) and post-RAIT samples (*p* = 0.002) from thyroid cancer patients, which is illustrated in the box-plots in Figure 4 and Figure 5, respectively.

**Figure 6:**
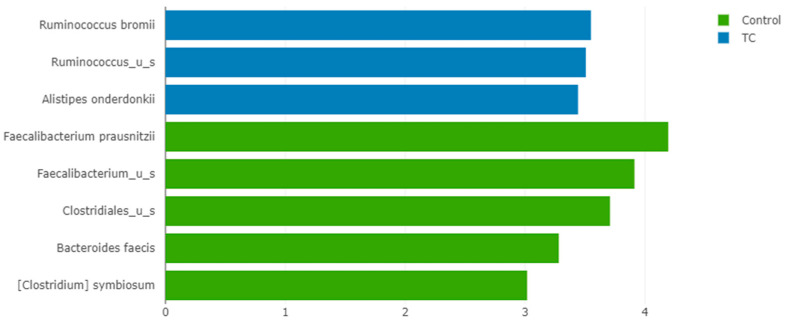
LEfSe chart representing the species that are significantly enriched in thyroid cancer patients (blue) and controls (green). All had LDA scores> 3 and *p* < 0.05.

Interestingly, LEfSe analysis found that *F. prausnitzii* was the most significantly enriched species in controls compared to thyroid cancer patients, with an LDA score > 4 and *p* < 0.001 (Figure 6).

### 3.4. Impact of Radioiodine Therapy on the Richness, Diversity, and Composition

Alpha diversity was not significantly different between the pre- and post-treatment sets of samples, using Chao1 and the Simpson and Shannon indexes (*p* = 0.456, *p* = 0.273, and *p* = 0.208, respectively). Regarding beta diversity, no separating trends between the bacterial communities in pre- and post-treatment groups were observed in terms of Jaccard distance and Bray–Curtis dissimilarity (*p* = 1.000 and *p* = 0.994, respectively).

No significant changes at the phylum and genus levels were found after RAIT. At the species level, through LEfSe analysis, we found that *Ruminococcus bicirculans* was enriched before treatment (LDA of 3.082, *p* = 0.005).

### 3.5. Impact of RAIT on the Relative Abundance of F. prausnitzii

Our results showed that the mean relative abundance of *F*. *prausnitzii* increased in the patients’ samples post-RAIT (1.76 ± 1.86%) compared to pre-RAIT samples (1.70 ± 1.96%) but without significance (*p* = 0.1485). The same pattern was observed in the absolute abundances (*p* = 0.1646). In two patients, *F. prausnitzii* was not detected in either sample.

**Figure 7:**
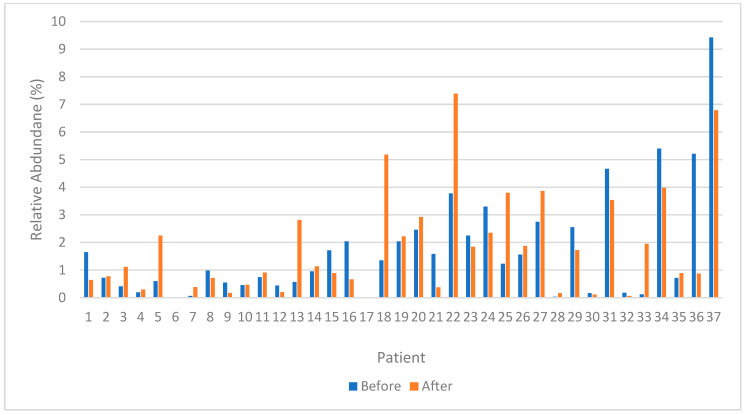
Differences in the relative abundance (%) of *Faecalibacterium prausnitzii* before and after treatment from each patient (n = 37).

When analyzing each patient, an increase in the relative abundance of *F*. *prausnitzii* after RAIT was seen in nineteen patients, and a decrease was observed in sixteen patients; in two, it was unchanged. The relative abundance of *F. Prausnitzii* pre- and post-treatment from each patient is summarized in Figure 7.

## 4. Discussion

To the best of our knowledge, our study was the first to identify the variation of the relative abundance of *F. prausnitzii* after RAIT using shotgun metagenomics. We also compared the gut microbiota from thyroid cancer patients with volunteers. *Faecalibacterium prausnitzii* is the most important and explored butyrate-producing bacteria and one of the most abundant species in the human gut microbiota.

### 4.1. Composition of the Gut Microbiota of Differentiated Thyroid Cancer Patients before Treatment: The Relative Abundance of F. prausnitzii Is Decreased in Thyroid Cancer Patients Compared to Controls

The gut microbiota in our population of thyroid cancer patients was characterized by a dominance of Bacteroidetes and Firmicutes at the phylum level, which are known to be the most prominent phyla of the human gut microbiota.

Previous studies have evaluated the gut microbiota composition of thyroid cancer patients and found a dominance of the genera *Prevotella*, *Roseburia*, *Coprococcus*, *Anaerostipes*, *Ruminococcus*, *Neisseria*, *Streptococcus*, and *Prophyromonas* [37]. Conversely, other studies found a decrease in *Prevotella* [27,30].

At the genus level, in both groups, we found a dominance of *Alistipes* and *Bacteroides*. However, the other most represented genera in thyroid cancer patients were *Phocaeicola*. In contrast, in the control group, *Faecalibacterium* and *Subdoligranulum* were, together with Alistipes and Bacteroides, the most represented genera, and both are butyrate-producing bacteria [47].

The connection between gut microbiota and thyroid diseases, including thyroid cancer, has been suggested by numerous studies [30,35,48,49]. In our study, the thyroid cancer patients (before and after RAIT) who also presented with temporary hypothyroidism had a significant reduction in the relative abundance of *F*. *prausnitzii* compared to controls. This result is in line with previous studies since these bacteria were also reported to be significantly less present in multiple other diseases, including Crohn’s disease [14,50,51], rheumatoid arthritis [52], ulcerative colitis [15,53], celiac disease [16], and obesity [17]. The low relative abundance may be due to hypothyroidism or thyroid cancer, or both. Previous research found a decrease in *F. prausnitzii* in hypothyroid Hashimoto’s thyroiditis [18]; contrarily, Lauritano et al. and Ishaq et al. found that hypothyroidism is associated with bacterial overgrowth development [54,55].

The link between thyroid diseases and short-chain fatty acids has also been explored. Zhang J et al. found that *Butyricimonas*, which produce short-chain fatty acids, are significantly lower in thyroid cancer patients [37]. Microbial products, particularly short-chain fatty acids, can serve as an energy source for enterocytes and, combined with thyroid hormones, may enhance enterocyte differentiation and strengthen intercellular tight junctions [48,56]. The microbiota may also play a role in I^−^ uptake and in the enterohepatic cycling of thyroid hormones [48].

Interestingly, Yu X et al. suggested that the loss of SCFAs-producing bacteria may promote the development of thyroid cancer [27]. They believe that a low abundance of *F. prausnitzii* may result in gut microbial dysbiosis before or after developing thyroid cancer [27]. Our study reinforces that thyroid cancer patients present with dysbiotic gut microbiota with a low abundance of one of the most important butyrate-producing bacterium.

### 4.2. Effects of Radioiodine Therapy

Previous studies in patients with different types of cancers exposed to abdominal and pelvic radiotherapy reported that there were decreases in the relative abundance of *F*. *prausnitzii* [41,42,43,44] and an increase in one study [45]. Previous similar studies that did not report changes in these bacteria used different, less sensitive analytical methodologies that may have failed to detect *F*. *prausnitzii*. The difference between our results and previously reported results from radiotherapy reinforces the position of the radiobiology European Association of Nuclear Medicine working group that “radiopharmaceutical treatments are well tolerated and that some regimens could undertreat patients” [57].

Our study shows a mixed response, with an overall increase in the relative abundance of *F*. *prausnitzii* after radioiodine therapy, but without significance. In most patients, we found an increase in *F*. *prausnitzii* after radioiodine therapy.

Lapiere et al. demonstrated that prophylactic treatment with *F. prausnitzii* protects the colonic mucosal barrier from radiation exposure by maintaining its functional integrity through the regulation of the intestinal permeability, the enhancement of epithelial self-renewal, and through the preservation of differentiated epithelial cells [58].

Ionizing radiation refers to high-energy radiation that can ionize atoms by removing electrons from them, causing damage to molecules and cells. Some molecular effects of ionizing radiation include DNA and protein damage, oxidative stress, and cell cycle arrest. Overall, the molecular effects of ionizing radiation depend on the type and dose of radiation and on the type of cells being exposed.

Ionizing radiation has several effects on gut microbiota, the most relevant being the induction of oxidative stress. It generates reactive oxygen and nitrogen species that can damage cellular components, impair cellular functions, and change the redox state of the intestinal environment. These changes affect microbial growth, survival, and metabolism and, therefore, alter the gut microbiota’s composition. In addition to the previously described effects, ionizing radiation is capable of modulating microbial gene expression as it can alter the expression of genes involved in functions including DNA repair, response to stress, and energy metabolism. These gene expression changes may lead to relevant alterations in microbial function and metabolism, which may affect host health. The gut microbiota is a complex and diverse ecosystem of microorganisms, and ionizing radiation can promote certain microbial species’ growth and suppress others.

When exposed to ionizing radiation, the intestine experiences damage to epithelial cells and disruption of the microbiota, leading to inflammation and increased susceptibility to infection. However, SCFAs produced by gut microbiota inhibit inflammation and promote the survival and repair of epithelial cells.

Short-chain fatty acids have been shown to reduce oxidative stress and DNA damage in intestinal cells and enhance the repair of damaged DNA. Additionally, short-chain fatty acids can improve intestinal barrier function, which protects against the infiltration of harmful bacteria and toxins.

Given the positive effects of short-chain fatty acids in metabolism and immune modulation, the increase in the relative abundance in most patients might indicate that these bacteria could be important in resolving radiation aggression.

## 5. Conclusions

In conclusion, in this study, we found a significant reduction in the relative abundance of *F. prausnitzii*, the most important butyrate-producing bacterium, in thyroid cancer patients compared to controls.

Regarding the effect of radioiodine therapy, it does not appear to negatively affect the human gut microbiota. Moreover, we found that most patients had a higher relative abundance of *F. prausnitzii* after treatment, which, in line with previous studies, could indicate that *F. prausnitzii*, due to its anti-inflammatory characteristics, could play an important role in resolving radiation aggression.

Further studies are needed to discern whether and to what extent *F*. *prausnitzii* effectively plays a role in the outcome of the patients.

## Figures and Tables

**Table 1:** Patient baseline characteristics (ND—not determined; SD—standard deviation).

Baseline Characteristics	n = 37
Age (mean, SD)	51 ± 17
Gender	
Male	11
Female	26
BMI (mean, SD)	28.3 ± 6.6
Concomitant diseases	
Diabetes	7
Cardiovascular diseases	16
Other (psoriasis, chronic kidney disease, hyperuricemia, gastritis, etc.)	8
Number of chronic medications (mean, SD)	3.1 ± 2.4
Tumor Type	
Follicular	1
Papillar	36
Clinical T stage	
T1	16
T2	13
T3	6
T4	1
ND	1
Clinical N stage	
Nx	25
N0	4
N1	8
Clinical M stage	
M0	34
M1	3
R	
R0	30
R1	5
R2	1
Age (mean, SD)	51 ± 17
Gender	
Male	11
Female	26
I-131 dose	
1110 MBq	7
3700 MBq	22
5550 MBq	8

## Data Availability

The data that support the findings of this study are available in Mendeley Data, V1 at http://doi.org/10.17632/3nfhkdmmzy.1, (accessed on 11 May 2023).

## Supplementary Materials

The following supporting information can be downloaded at: https://www.mdpi.com/article/10.3390/nu15122680/s1, Table S1: Relative abundance Phylum-level before/after RAIT and volunteers; Table S2: Genus level-relative abundance before/after RAIT; Table S3: Relative abundance genus level in volunteers; Table S4: Species relative abundance from controls; Table S5: Species-level relative abundance before/after RAIT; Table S6: LefSe analysis Controls versus Thyroid cancer; Table S7: Change in absolute abundance phylum level before/after RAIT. after the Bonferroni correction; Table S8: Change in absolute abundance genus level before/after RAIT. *p*-values after the Bonferroni correction; Table S9: Change in absolute abundance species-level before/after RAIT. *p*-values after the Bonferroni correction; Table S10: Change in relative abundance phylum level before/after RAIT. *p*-values after the Bonferroni correction; Table S11: Change in relative abundance genus-level before/after RAIT. *p*-values after the Bonferroni correction; Table S12: Change in relative abundance species-level before/after RAIT. *p*-value after the Bonferroni correction.
