# Supplementary material for: Faecalibacterium prausnitzii in Differentiated Thyroid Cancer Patients Treated with Radioiodine
Source: Nutrients. 2023 Jun 8;15(12):2680. doi: 10.3390/nu15122680 (PMC10301062; doi:10.3390/nu15122680)
Supplement: Supplementary file 1 [file nutrients-15-02680-s001.zip › nutrients-2420599-supplementary.pdf]

Supplementary materials

Table S1 - Relative abundance Phylum-level before/after RAIT and volunteers

	**Mean ± SD**		
**Phylum**	**Before RAIT**	**After RAIT**	**Volunteers**
**Firmicutes**	46.25±17.64%	43.29±17.81%	50.78±14.88%
**Bacteroidetes**	39.26±21.06%	42.31±19.72%	33.31±11.78%
**Actinobacteria**	8.66±13.27%	4.93±5.85%	6.20±5.38%
**Proteobacteria**	5.22±8.25%	5.92±10.05%	8.62±12.31%
**Verrucomicrobia**	2.54±6.77%	3.15±7.21%	0.98±1.99%

Table S2 - Genus level- relative abundance before/after RAIT

		Mean	SD
Akkermansia	before	1.72%	4.31%
	after	3.15%	7.21%
Alistipes	before	10.03%	6.40%
	after	10.90%	7.02%
Bacteroides	before	13.25%	12.94%
	after	13.84%	9.93%
Phocaeicola	before	5.11%	4.27%
	after	6.48%	5.86%
Bifidobacterium	before	4.27%	6.46%
	after	2.79%	4.04%
Blautia	before	1.37%	1.45%
	after	0.93%	1.05%
Lachnospiraceae_u_g	before	3.34%	3.49%
	after	3.15%	2.53%
Firmicutes_u_g	before	4.23%	5.22%
	after	3.39%	3.98%
Clostridium	before	1.73%	1.58%
	after	1.74%	2.38%
Ruminococcaceae_u_g	before	3.38%	2.68%
	after	3.67%	3.46%
Dialister	before	1.08%	2.22%
	after	1.67%	4.37%
Dorea	before	1.30%	2.17%
	after	0.94%	1.96%
Escherichia	before	2.06%	3.97%

	after	3.67%	9.62%
Faecalibacterium	before	2.78%	3.04%
	after	3.02%	3.19%
Gemmiger	before	2.66%	5.39%
	after	1.62%	1.91%
Lactobacillus	before	0.80%	1.42%
	after	0.40%	0.91%
Parabacteroides	before	3.96%	3.21%
	after	4.09%	3.71%
Prevotella	before	3.76%	7.92%
	after	3.67%	9.33%
Roseburia	before	2.38%	3.34%
	after	1.74%	2.06%
Ruminococcus	before	2.25%	1.82%
	after	1.82%	2.48%
Subdoligranulum	before	3.28%	3.40%
	after	4.21%	6.69%

Table S3 - Relative abundance genus level in volunteers

	Mean	SD
Akkermansia	0.98%	1.99%
Alistipes	11.04%	4.90%
Bacteroides	10.00%	6.63%
Phocaeicola	4.02%	3.19%
Bifidobacterium	3.64%	4.12%
Blautia	1.27%	1.25%
Lachnospiraceae_u_g	3.70%	2.03%
Firmicutes_u_g	4.99%	5.77%
Clostridium	2.58%	2.02%
Ruminococcaceae_u_g	3.16%	3.38%
Dialister	1.93%	2.49%
Dorea	0.62%	0.64%
Escherichia	2.51%	4.47%
Faecalibacterium	7.06%	3.02%
Gemmiger	3.39%	2.73%
Lactobacillus	0.06%	0.13%
Parabacteroides	2.58%	2.27%
Prevotella	2.78%	4.18%
Roseburia	1.80%	1.64%
Ruminococcus	0.96%	0.98%
Subdoligranulum	5.03%	5.89%

Table S4 – Species relative abundance from controls

	Mean	SD
Escherichia coli	2.49%	4.44%
Akkermansia muciniphila	0.93%	1.92%
Bifidobacterium longum	1.66%	1.86%
Bifidobacterium adolescentis	0.50%	0.59%
Subdoligranulum_u_s	5.00%	5.90%
Ruminococcus_u_s	0.47%	0.37%
Ruminococcaceae_u_s	3.16%	3.38%
Ruminococcus bromii	0.07%	0.22%
Gemmiger formicilis	3.34%	2.76%
Firmicutes_u_s	4.99%	5.77%
Faecalibacterium prausnitzii	4.50%	2.21%
Faecalibacterium_u_s	2.55%	1.06%
Clostridium_u_s	2.02%	1.68%
Blautia_u_s	1.12%	1.20%
Prevotella copri	2.10%	3.88%
Phocaeicola vulgatus	2.34%	2.51%
Phocaeicola dorei	0.89%	1.12%
Parabacteroides_u_s	0.97%	1.00%
Bacteroides_u_s	3.72%	2.50%
Bacteroides uniformis	3.08%	2.52%
Alistipes putredinis	4.39%	2.51%
Alistipes shahii	1.35%	1.21%
Alistipes_u_s	1.54%	1.31%

Table S5 - Species-level relative abundance before/after RAIT

		Mean	SD
Escherichia coli	before	1.95%	3.95%
	after	3.34%	9.55%
Akkermansia muciniphila	before	1.62%	4.33%
	after	3.00%	7.18%
Bifidobacterium longum	before	0.77%	1.13%
	after	0.61%	0.98%
Bifidobacterium adolescentis	before	2.44%	5.17%
	after	1.16%	1.86%
Subdoligranulum_u_s	before	3.27%	3.39%
	after	4.21%	6.69%
Ruminococcus_u_s	before	1.15%	0.87%
	after	1.04%	1.39%
Ruminococcaceae_u_s	before	3.38%	2.68%
	after	3.67%	3.46%
Ruminococcus bromii	before	0.80%	0.85%
	after	0.67%	1.15%
Gemmiger formicilis	before	2.66%	5.39%
	after	1.61%	1.91%
Firmicutes_u_s	before	4.23%	5.22%
	after	3.39%	3.98%
Faecalibacterium prausnitzii	before	1.70%	1.96%
	after	1.76%	1.86%
Faecalibacterium_u_s	before	1.08%	1.15%
	after	1.25%	1.50%
Clostridium_u_s	before	1.40%	1.59%
	after	1.42%	2.28%
Blautia_u_s	before	0.83%	0.83%
	after	0.58%	0.64%
Prevotella copri	before	2.72%	6.48%
	after	2.25%	6.54%
Phocaeicola vulgatus	before	2.30%	2.38%
	after	3.39%	3.43%
Phocaeicola dorei	before	1.55%	1.75%
	after	1.75%	1.75%
Parabacteroides_u_s	before	1.87%	1.86%
	after	1.96%	2.23%
Bacteroides_u_s	before	5.15%	4.84%
	after	5.46%	3.84%
Bacteroides uniformis	before	3.87%	4.63%
	after	3.74%	3.23%
Alistipes putredinis	before	3.53%	2.92%
	after	3.85%	3.44%

Alistipes shahii	before	0.99%	1.03%
	after	1.16%	1.18%
Alistipes_u_s	before	1.73%	1.47%
	after	1.99%	1.64%

Table S6 – LefSe analysis Controls versus Thyroid cancer

Feature	Log Highest Mean	Cohort	Lda	P-Value
Faecalibacterium prausnitzii	4.653579129618571	Control	4.19516870809131	0.000247119841581
Faecalibacterium_u_s	4.406887556045944	Control	3.913419462969283	0.0013830875381884
Clostridiales_u_s	4.220074400520429	Control	3.7090532118609154	0.0399084228452192
Ruminococcus_u_s	4.061622015937191	TC	3.507459385718969	0.0074205407929063
Ruminococcus bromii	3.905062379064919	TC	3.549936798276648	0.003091664128723
Bacteroides faecis	3.786637689700976	Control	3.282148053378805	0.0206167465848876
[Clostridium] symbiosum	3.367560952570651	Control	3.017963388398882	0.0436778750197986
Alistipes onderdonkii	3.919430809994788	TC	3.442509789012845	0.0289901213340677

Table S7 –Change in absolute abundance phylum level before/after RAIT. after the Bonferroni correction

	Thyroid cancer patients	
Phylum	Change in absolute abundance (%)[a]	p-value[b.c]
Actinobacteria	-43.3 [-69.2 to 52.5]	1.000
Proteobacteria	14.9 [-52.6 to 216.7]	0.550
Bacteroidetes	5.2 [-35.5 to 88.7]	0.960
Firmicutes	-4.7 [-51.4 to 37.7]	1.000
Verrucomicrobia	-8.6 [-87.7 to 52.2]	1.000

a – median [IQR]

b – Bonferroni correction

c – Wilcoxon signed ranks test

Table S8 – Change in absolute abundance genus level before/after RAIT. p-values after the Bonferroni correction

	Thyroid cancer	
Genus	Change in absolute abundance (%)[a]	p-value[b.c]
Dorea	-28.6 [-79.7 to 69.5]	1.000
Phocaeicola	4.2 [-46 to 99.9]	1.000
Lactobacillus	-63 [-93.8 to -6.8]	0.105
Firmicutes_u_g	-42.9 [-69.3 to 54.2]	1.000
Prevotella	29.3 [-71.1 to 202]	1.000
Ruminococcus	-23.6 [-79.4 to 104]	1.000
Clostridium	-31.2 [-63 to 32.8]	1.000
Faecalibacterium	-19.7 [-51 to 96.5]	1.000
Ruminococcaceae_u_g	-9.6 [-48.6 to 74.7]	1.000

Akkermansia	-8.6 [-87.7 to 52.2]	1.000
Bifidobacterium	-52.6 [-81.3 to 7.7]	1.000
Blautia	-47.7 [-80.4 to 85.2]	1.000
Subdoligranulum	-30.3 [-65 to 65.9]	1.000
Gemmiger	-33.2 [-54 to 70.5]	1.000
Roseburia	-29.2 [-68 to 144.2]	1.000
Escherichia	25.1 [-91.9 to 150.8]	1.000
Bacteroides	16.3 [-28.5 to 76.1]	1.000
Lachnospiraceae_u_g	-25.6 [-61.3 to 110.3]	1.000
Dialister	-26.7 [-92.3 to 11.1]	1.000
Alistipes	-11.6 [-51.2 to 127.7]	1.000
Parabacteroides	-7.5 [-46.1 to 98.4]	1.000

Table S9 – Change in absolute abundance species-level before/after RAIT. p-values after the Bonferroni correction

	Thyroid cancer	
Species	Change in absolute abundance (%)[a]	p-value[b.c]
Blautia_u_s	-48.3 [-81.9 to 38.6]	1.000
Ruminococcus_u_s	-23.7 [-70.3 to 183.1]	1.000
Ruminococcus.bromii	-67 [-97.1 to -5.4]	0.322
Firmicutes_u_s	-42.9 [-69.3 to 54.2]	1.000
Alistipes.putredinis	2.1 [-41.8 to 133.8]	1.000
Parabacteroides_u_s	-15.7 [-62.7 to 87.6]	1.000
Bacteroides.uniformis	1.5 [-59.7 to 68.1]	1.000
Phocaeicola.vulgatus	-8.3 [-42.5 to 108.7]	1.000
Akkermansia.muciniphila	-8.2 [-99.6 to 64]	1.000
Alistipes.shahii	3.2 [-41.3 to 53.1]	1.000
Escherichia.coli	41.5 [-86.4 to 297.8]	1.000
Faecalibacterium_u_s	-22.9 [-49.8 to 59]	1.000
Phocaeicola.dorei	-2.6 [-46.4 to 73.1]	1.000
Bifidobacterium.longum	-63.4 [-92.8 to -9.5]	0.345
Faecalibacterium.prausnitzii	-20.8 [-46.9 to 131.5]	1.000
Subdoligranulum_u_s	-30.3 [-64.9 to 65.7]	1.000
Alistipes_u_s	-6.5 [-44.1 to 119.4]	1.000
Clostridium_u_s	-39.9 [-76.8 to 76.9]	1.000
Ruminococcaceae_u_s	-9.6 [-48.6 to 74.7]	1.000
Prevotella.copri	-58.3 [-88.6 to 46.4]	1.000
Gemmiger.formicilis	-33.2 [-63.9 to 70.5]	1.000
Bifidobacterium.adolescentis	-66.6 [-88.7 to -18.8]	1.000
Bacteroides_u_s	-0.9 [-48.8 to 82.2]	1.000

Table S10 – Change in relative abundance phylum level before/after RAIT. p-values after the Bonferroni correction

	Thyroid cancer	
Phylum	Change in **relative** abundance (%)[a]	p-value[b.c]

Actinobacteria	-28.2 [-67.6 to 34.5]	1.000
Proteobacteria	-0.2 [-42 to 158.2]	0.810
Bacteroidetes	5.7 [-16.9 to 49]	1.000
Firmicutes	-10.2 [-28.8 to 23.8]	1.000
Verrucomicrobia	2.3 [-92.3 to 94.3]	1.000

a – median [IQR]

b – Bonferroni correction

c – Wilcoxon signed ranks test

Table S11 – Change in relative abundance genus-level before/after RAIT. p-values after the Bonferroni correction

	Thyroid cancer	
Genus	Change in **relative** abundance (%)[a]	p-value[b.c]
Dorea	-25.7 [-76 to 94.9]	1.000
Phocaeicola	40.2 [-34.6 to 89]	0.966
Lactobacillus	-67.9 [-93.3 to 4.3]	0.231
Firmicutes_u_g	-41.5 [-71.7 to 70.5]	1.000
Prevotella	-25.8 [-60.7 to 167.1]	1.000
Ruminococcus	-31.9 [-70.2 to 68.4]	1.000
Clostridium	-24.7 [-61.2 to 37.5]	1.000
Faecalibacterium	4.6 [-41 to 52.7]	1.000
Ruminococcaceae_u_g	-9.1 [-36.3 to 44.6]	1.000
Akkermansia	2.8 [-89.1 to 94.3]	1.000
Bifidobacterium	-40.3 [-80 to 29.9]	1.000
Blautia	-49 [-73.9 to 44.3]	1.000
Subdoligranulum	-14.3 [-47.2 to 58.2]	1.000
Gemmiger	-17.6 [-56.9 to 62.1]	1.000
Roseburia	-17.7 [-63.3 to 66.8]	1.000
Escherichia	-11.9 [-92.3 to 198.7]	1.000
Bacteroides	15.4 [-28.4 to 63.7]	1.000
Lachnospiraceae_u_g	8.4 [-61.9 to 85.3]	1.000
Dialister	-30 [-98.2 to 8.3]	1.000
Alistipes	-6.1 [-38.2 to 93.4]	1.000
Parabacteroides	-1.6 [-42.4 to 79.5]	1.000

Table S12 – Change in relative abundance species-level before/after RAIT. p-value after the Bonferroni correction

	Thyroid cancer	
Species	Change in absolute abundance (%)[a]	p-value[b.c]
Blautia_u_s	-32.3 [-76.3 to 39]	1.000
Ruminococcus_u_s	-27.3 [-70 to 66.7]	1.000
Ruminococcus.bromii	-66.3 [-92.7 to 17.1]	0.966
Firmicutes_u_s	-41.5 [-71.7 to 70.5]	1.000
Alistipes.putredinis	1.4 [-32.5 to 108.9]	1.000

Parabacteroides_u_s	-13.5 [-52.9 to 89.4]	1.000
Bacteroides.uniformis	-18.3 [-46.3 to 66.2]	1.000
Phocaeicola.vulgatus	29.9 [-31.3 to 93.5]	1.000
Akkermansia.muciniphila	3 [-99.5 to 116.1]	1.000
Alistipes.shahii	6.6 [-16.6 to 40.6]	1.000
Escherichia.coli	-14.9 [-87.1 to 323.1]	1.000
Faecalibacterium_u_s	4 [-32.9 to 67.3]	1.000
Phocaeicola.dorei	21.3 [-47.6 to 53.2]	1.000
Bifidobacterium.longum	-52.7 [-86.4 to 15.9]	1.000
Faecalibacterium.prausnitzii	7.5 [-31.9 to 75.3]	1.000
Subdoligranulum_u_s	-13.9 [-47.1 to 58]	1.000
Alistipes_u_s	2 [-27.5 to 106.2]	1.000
Clostridium_u_s	-10.1 [-75 to 48.4]	1.000
Ruminococcaceae_u_s	-9.1 [-36.3 to 44.6]	1.000
Prevotella.copri	-49 [-88.5 to 1.6]	1.000
Gemmiger.formicilis	-17.6 [-66.2 to 62.1]	1.000
Bifidobacterium.adolescentis	-43.5 [-83.4 to 21]	1.000
Bacteroides_u_s	12 [-33.6 to 61.9]	1.000
